# Parents’ information needs and influential factors when making decisions about TNF-α inhibitors

**DOI:** 10.1186/s12969-016-0113-5

**Published:** 2016-09-15

**Authors:** Ellen A. Lipstein, Daniel J. Lovell, Lee A. Denson, Sandra C. Kim, Charles Spencer, Maria T. Britto

**Affiliations:** 1 Division of Adolescent and Transition Medicine, Cincinnati Children’s Hospital Medical Center, 3333 Burnet Ave, Cincinnati, OH 45229 USA; 2 James M. Anderson Center for Health Systems Excellence, Department of Pediatrics, Cincinnati Children’s Hospital Medical Center, Cincinnati, USA; 3 Division of Rheumatology, Department of Pediatrics, Cincinnati Children’s Hospital Medical Center, Cincinnati, USA; 4 Division of Gastroenterology, Hepatology and Nutrition, Department of Pediatrics, Cincinnati Children’s Hospital Medical Center, Cincinnati, USA; 5 Division of Gastroenterology, Hepatology and Nutrition, Department of Pediatrics, Nationwide Children’s Hospital, Columbus, USA; 6 Division of Rheumatology, Department of Pediatrics, Nationwide Children’s Hospital, Columbus, USA; 7 University of Cincinnati College of Medicine, Cincinnati, USA; 8 The Ohio State College of Medicine, Columbus, USA

## Abstract

**Background:**

Parents struggle when making treatment decisions for children with arthritis or other chronic conditions. Understanding their decision-making process is an essential step towards improving the decision-making experience. The objective of this study was to describe parents’ information needs and the influences on their decision making about treatment with TNF-α inhibitors.

**Methods:**

Survey domains were developed based on qualitative data and cognitive interviewing. We mailed the survey to parents of children with juvenile idiopathic arthritis or inflammatory bowel disease who had initiated treatment with TNF-α inhibitors in the prior 2 years. Data were analyzed using descriptive and non-parametric statistics.

**Results:**

Survey response rate was 54.9 %. Each item had <2 % missing responses. Parents used an array of information sources when deciding about treatment with TNF-α inhibitors. Resources other than their child’s specialist were most often used to increase confidence in parents’ decisions or because they wanted to know more about other people’s experiences being treated with TNF-α inhibitors, rather than due to a lack of understanding. All but two (cost and route of administration) of the influential decision factors were very or extremely important to the majority of participants with factors related to long-term side effects, treatment efficacy, and disease impact being most important.

**Conclusions:**

This study describes parents’ information needs and influential factors in treatment decision making. Results suggest that future work should be aimed at helping families weigh risks and benefits, such as through decision support interventions, as well as developing opportunities to include people beyond the family and physician in the decision-making process.

**Electronic supplementary material:**

The online version of this article (doi:10.1186/s12969-016-0113-5) contains supplementary material, which is available to authorized users.

## Background

Shared decision making is a key element of family-centered care [1]. In order to participate in decision making, parents need to be informed and prepared to weigh the risks and benefits of treatment options. In the setting of chronic conditions, parents often lack information and may later reconsider difficult treatment decisions [2–6]. Decisions about high-risk or lengthy treatments may be particularly challenging and stressful for parents [7, 8].

For families of children and adolescents with juvenile idiopathic arthritis (JIA) or inflammatory bowel disease (IBD), our prior, qualitative research found that the decision to start TNF-α inhibitors can be particularly challenging [7, 9, 10] due to the need to balance disease severity with side-effect risks [11–13]. As such, parents struggle to weigh the pros and cons. This struggle continues after the decision is made, as they continue to worry about the potential consequences of their decision. In order to help them make this challenging decision, parents report seeking information from diverse sources, including the internet and social contacts, [7] though the reasons they seek this information are unknown. Our prior research further suggested that the decision process, including the information needs and decision-making influences, may differ between parents of children with JIA and parents of children with IBD [7, 10].

These qualitative studies provided insight into parents’ decision-making experiences but were not intended to be generalizable. Moving forward we wanted to ensure that future interventions to address parents’ decision-making needs and improve their experience are designed with a full understanding of the influential factors in parents’ decisions. Although others have assessed parents’ information needs related to specific diseases [14–17] and general measures have been used to study the parent-provider interaction, [18–20] there were no existing measures or surveys that matched our desire to be able to describe the details of parents’ decision-making about TNF-α inhibitors.

## Methods

### Survey development

Survey questions were developed based on our prior qualitative interviews with families who had made decisions about treatment with TNF-α inhibitors [7, 10] and established models of shared decision making [21–23]. Specifically we sought to develop questions that captured elements of the decision-making experience that occurred both within and outside of the clinical encounter.

We generated a large pool of candidate questions (*n* = 51) and then reviewed them systematically, comparing each to our aims and to other candidate questions, to identify those that best fit the aims of our study without being duplicative [24, 25]. The remaining pool of questions was reviewed by clinicians (*n* = 2), parents (*n* = 2) and researchers with expertise in survey methods (*n* = 4). During this process, questions underwent several iterations to achieve face validity, the point at which all members of the review team were satisfied that the questions were complete and not duplicative in addressing our aims. These questions were divided into two survey domains: Parents’ Information Needs and Influential Decision Factors.

For each domain, the questions were tested via in-person cognitive interviews with parents of children with either JIA or IBD who had made a decision to start treatment with TNF-α inhibitors. Parents were recruited via referral from their child’s rheumatologist or gastroenterologist with the goal of including parents with diverse demographic characteristics, particularly in relation to parent education and child’s age. Recruitment continued until no substantial changes were needed in three consecutive interviews. After obtaining written consent, participants completed a “three-step test-interview” [26] in which they first answered the survey while describing out loud their thought process. Next, the interviewer asked questions related to her observations (for example, why a participant paused while answering a question). Finally, questions aimed at eliciting their experiences and opinions were asked. In this final step, participants were asked to complete tasks such as rephrasing questions in their own words, explaining the differences between similar questions and telling about aspects of decision making that they felt were not assessed in the survey. In analyzing the interviews the study team looked for consistency between verbal explanations and responses chosen, as well as accuracy in rephrasing questions in the participant’s own words. Parents were compensated $30 for participating in cognitive interviewing. The survey was revised, as described in the results, after every three interviews.

#### Final domains

Parents’ Information Needs focused on how and why information is obtained (Tables 2 and 3). We asked about information resources using the following question: “When deciding about treatment with TNF-α inhibitors, how important was information from each of the following people or resources…?” This was followed by a list of information sources. Response options included “didn’t use”, “not at all important”, “a little important”, “somewhat important”, “very important” and “extremely important”. We then asked “How often did you use sources of information other than your child’s [gastroenterology/rheumatology] provider…?” followed by a list of possible reasons. Response options in this section were “never”, “rarely”, “occasionally”, “pretty often” and “very often”.

Influential Decision Factors asked parents, “In making the decision about treatment with biologics how important was…?” followed by a list of potential decision factors (Table 4). For most items the response options were “not at all important”, “a little important”, “somewhat important”, “very important” and “extremely important”. For questions related to specific potential side effects there was an additional response option of “didn’t know about this.”

Parents received a survey that was specific to the disease their child has, either JIA or IBD (see Additional file 1). For example, questions referred to rheumatology for parents of children with JIA and gastroenterology for parents of children with IBD.

### Sample

Parents of children with JIA or IBD who initiated treatment with TNF-α inhibitors in the prior two years, at either of two Midwestern children’s hospitals that serve both local and national/international patients, were identified using disease registries and electronic medical records. One site chose to verify the pool of participants by hand due to recent changes in the structure of their disease registry. Parents of children who had a co-morbidity that could also be treated with a biologic were excluded, as were parents who had participated in cognitive interviewing. Patients with different names but the same mailing address were assumed to be siblings. In the case of siblings, we selected the sibling who had most recently started TNF-α inhibitors and a survey was sent addressed to that patient’s parents.

### Survey procedures

Collaborating clinics mailed potential participants a letter explaining the survey and how to opt-out of participation. Parents who did not opt-out were then mailed a survey along with a cover letter, a postage-paid return envelope and a $2 bill. Completion of the survey constituted consent to participate. Participants could decline to participate by calling the study coordinator or returning a blank survey. A reminder postcard was sent a week later, followed by a second copy of the survey. Non-responders were then contacted via telephone and offered an opportunity to complete the survey over the phone [27]. Three parents completed the survey over the phone.

This study was reviewed and approved by the Cincinnati Children’s Hospital Medical Center Institutional Review Board which served as the institutional review board of record for the study.

### Data analysis

We used non-parametric statistics to assess for differences, in demographics or responses to individual questions, based upon disease. In assessing the percent of responses missing for each question, we considered multiple responses or an unclear response, such as a mark between 2 response options, to be missing for that question. We addressed multiple comparisons by considering only *p*-values <0.01 as significant. Descriptive statistics were used for reporting response distributions. SAS 9.3 (SAS Institute Inc., Cary, NC) was used for all analyses.

## Results

### Survey development

Ten parents, five from each condition, participated in cognitive interviews. Most changes were only minor word or formatting alterations to improve readability. We observed no significant difficulty in comprehension of any question stem. For example, with the stem, “in making the decision about treatment with biologics, how important was…?” parents were able to correctly re-phrase the question in their own words for both risks and benefits.

A few questions were clarified through the interviewing process. For example, we found that parents distinguished between the ways treatment affects their child’s daily life and the ways JIA or IBD affects their daily life. Also, we asked parents both how important “the likelihood that treatment with biologics would work for your child” was and how important “the success rate of treatment with biologics?” was, intending to use one question to assess the importance of data to parents’ decisions. However, parents consistently viewed these as two different questions; the first related to the chance their specific child would improve on TNF-α inhibitors and the second related to the overall success in the population. For this reason, both questions were retained in the final survey.

When the final survey was fielded, many questions had no missing responses. Those that had missing responses, for reasons other than the item not being applicable, were each missing responses from <2 % of respondents.

### Survey demographics

The final survey had a total of 201 respondents (response rate 54.9 %). Children of responding parents had a mean age of 13.9 years and had started TNF-α inhibitors a mean of 17.4 months prior to the survey. 42.8 % of respondents had a child with JIA. On average children with JIA were younger than those with IBD, more likely to be female and had started TNF-α inhibitors longer ago. Other demographics were similar and all are listed in Table 1.

**Table 1:** Demographics

Characteristic	Juvenile Idiopathic Arthritis (*n* = 86)	Inflammatory Bowel Disease (*n* = 115)	Total (*n* = 201)	*P* Value*
Patient age, mean (SD), y	12.0 (4.9)	15.4 (3.1)	13.7 (4.0)	<0.001
Months since starting treatment, mean (SD)	20.3 (8.1)	15.5 (7.4)	17.9 (7.8)	<0.001
Patient sex, n (%)				0.003
Male	30 (34.9)	64 (56.1)	94 (47)	
Female	56 (65.1)	50 (43.9)	106 (53)	
Patient race, n (%)				0.32
White	77 (90.6)	98 (86.0)	175 (87.9)	
Black/African American	5 (5.9)	7 (6.1)	12 (6.0)	
Asian/American Indian/Pacific Islander	0 (0)	3 (2.6)	3 (1.5)	
Mixed	3 (3.5)	3 (2.6)	6 (3.1)	
Other	0 (0)	3 (2.6)	3 (1.5)	
Patient ethnicity, n (%)				0.25
Hispanic	5 (5.8)	3 (2.6)	8 (4.0)	
Non-Hispanic	81 (94.2)	112 (97.4)	193 (96.0)	
Respondent relationship to patient				0.37
Mother	78 (91.8)	98 (85.2)	176 (88.0)	
Father	6 (7.1)	15 (13.0)	21 (10.5)	
Legal Guardian	1 (1.2)	2 (1.7)	3 (1.5)	
Respondent education, n (%)				0.06
No college	13 (15.7)	9 (8.2)	22 (11.4)	
Some college	22 (26.5)	23 (20.9)	45 (23.3)	
College degree	25 (30.1)	47 (42.7)	72 (37.3)	
Post-graduate degree	21 (25.3)	30 (27.3)	51 (26.4)	
Other	2 (2.4)	1 (0.9)	3 (1.6)	

*p-value comparing characteristics between IBD and JIA

Overall there were few statistical differences when comparing responses from parents whose children have JIA to those whose children have IBD. Therefore results from both diseases are grouped, with any between group differences discussed in the text.

### Parents’ information needs

The most commonly used information sources when parents were deciding about treatment with TNF-α inhibitors were their child’s rheumatologist or gastroenterologist (hereafter referred to as specialists), the nurse in the specialist’s clinic, materials from the makers of TNF-α inhibitors, and the internet, with the first three also being the most important sources. Of the 62.7 % of parents who used their child’s primary care provider as a source of information, 19.8 % reported that the primary care provider was an extremely important source of information and 23.0 % found them to be very important. Of the 66.0 % of parents who used friends or family members as a source of information, 9.1 % reported that they were extremely important and 23.5 % very important. We found that significantly more parents of children with JIA, compared to IBD, used the pharmacist as an information source (*p* < 0.001). 99.0 % of respondents found information from the specialist to be very or extremely important. More than 85 % also used the specialist’s nurse as an information source, with 64.6 % finding information from the nurse to be very or extremely important. Information from the internet or materials from makers of TNF-α inhibitors was very or extremely important to only 41.9 % and 54.2 % of respondents who used those sources (Table 2).

**Table 2:** Parents’ information needs: use and importance of information sources

		Among those using the resource				
	Didn’t Use N (%)	Not at all Important N (%)	A little Important N (%)	Somewhat Important N (%)	Very Important N (%)	Extremely Important N (%)
Your child’s [rheumatology/gastroenterology] provider	0 (0)	0 (0)	1 (0.5)	1 (0.5)	28 (14.0)	170 (85.0)
Your child’s primary care provider	75 (37.3)	22 (17.5)	25 (19.8)	25 (19.8)	29 (23.0)	25 (19.8)
The [rheumatology/gastroenterology] nurses	29 (14.4)	9 (5.2)	13 (7.6)	39 (22.7)	66 (38.4)	45 (26.2)
Your pharmacist	95 (47.3)	25 (23.6)	21 (19.8)	24 (22.6)	22 (20.8)	14 (13.2)
Friends and family members	68 (34.0)	22 (16.7)	26 (19.7)	41 (31.1)	31 (23.5)	12 (9.1)
Parents of children with [arthritis/ulcerative colitis or Crohns disease]	86 (42.8)	9 (7.8)	27 (23.5)	25 (21.7)	39 (33.9)	15 (13.0)
Materials, such as brochures or videos, from the makers of biologics	47 (23.5)	4 (2.6)	20 (13.1)	46 (30.1)	51 (33.3)	32 (20.9)
Advertisements	91 (45.5)	45 (41.3)	31 (28.4)	18 (16.5)	9 (8.3)	6 (5.5)
The internet	36 (17.9)	10 (6.1)	29 (17.6)	57 (34.6)	44 (26.7)	25 (15.2)

We then asked respondents the specific reasons they used sources of information other than their child’s specialist. The most frequent reasons for using other information sources were to increase confidence in their decision and because they wanted to know about other people’s experiences being treated with TNF-α inhibitors. Few parents used sources other than the specialist because they did not understand the information from the specialist or did not remember what was said during the visit (Table 3).

**Table 3:** Parents’ information needs: reasons for using information sources

	Never N (%)	Rarely N (%)	Occasionally N (%)	Pretty Often N (%)	Very Often N (%)
To prepare for a [rheumatology/gastroenterology] visit	44 (22.0)	37 (18.5)	73 (36.5)	33 (16.5)	13 (6.5)
Because you did not understand the information from the [rheumatology/gastroenterology] provider	86 (43.0)	69 (34.5)	38 (19.0)	6 (3.0)	1 (0.5)
Because you wanted more information than the [rheumatology/gastroenterology] provider gave you	26 (12.9)	39 (19.4)	73 (36.3)	37 (18.4)	26 (12.9)
Because you did not remember what was said during the visit about treatment with biologics	72 (36.0)	60 (30.0)	51 (25.5)	14 (7.0)	3 (1.5)
Because you wanted to know about other people’s experiences being treated with biologics	26 (13.0)	24 (12.0)	70 (35.0)	50 (25.0)	30 (15.0)
To increase your confidence in your decision	18 (9.0)	19 (9.5)	67 (33.5)	50 (25.0)	46 (23.0)

### Influential decision factors

All potential decision factors, except out of pocket treatment costs and the fact that TNF-α inhibitors are only available as a shot or infusion, were very or extremely important to more than half of respondents. The treatment factors which the most parents (>95 %) considered to be very or extremely important to the decision were decreasing their child’s symptoms, the impact of disease on their child’s day to day life, the likelihood that treatment would work for their child and the overall success rate of treatment with TNF-α inhibitors. Regarding the importance of side effects to parents’ decisions, avoiding long-term side effects from TNF-α inhibitors, the possible risk of cancer associated with treatment, and the effect of treatment on the child’s immune system were very or extremely important to the most parents, although 4.5, 6.0 and 0.5 % of parents respectively did not know about each of those (Table 4). The only risk or benefit with a statistically different response between diseases was the location, such as home or hospital, of treatment which was very or extremely important to more parents of patients with JIA than with IBD (*p* <0.001).

**Table 4:** Influential decision factors

	Not at all Important N (%)	A little Important N (%)	Somewhat Important N (%)	Very Important N (%)	Extremely Important N (%)	Didn’t know about this N (%)^a^
Logistics						
The results of medical tests, such as blood work or x-rays	6 (3.0)	5 (2.5)	21 (10.5)	65 (32.3)	104 (51.7)	-
How long your child would be treated with biologics	9 (4.6)	9 (4.6)	34 (17.2)	64 (32.3)	82 (41.4)	-
The ability to change your child’s treatment or try other treatments in the future	11 (5.5)	10 (5.0)	49 (24.6)	66 (33.2)	63 (31.7)	-
The fact that biologics are only available as a shot or IV infusion	34 (16.9)	32 (15.9)	50 (24.9)	34 (16.9)	51 (25.4)	-
The location, such as home or hospital, where your child could receive treatment with biologics	23 (11.4)	30 (14.9)	47 (23.4)	49 (24.4)	52 (25.9)	-
Your child’s age	21 (10.5)	22 (11.0)	35 (17.4)	58 (28.9)	65 (32.3)	-
The out-of-pocket cost of treatment	39 (19.4)	26 (12.9)	48 (23.9)	40 (19.9)	48 (23.9)	-
Risks and benefits						
Decreasing the symptoms your child was having at the time	0 (0)	0 (0)	2 (1.0)	34 (16.9)	165 (82.1)	-
Side effects your child had experienced with other kinds of medications^b^	16 (10.5)	13 (8.6)	23 (15.1)	38 (25.0)	62 (40.8)	-
The ways [arthritis/IBD] affects your child’s day to day life	1 (0.5)	1 (0.5)	4 (2.0)	55 (27.8)	137 (69.2)	-
The ways [arthritis/IBD] treatment affects your child’s day to day life	4 (2.0)	11 (5.5)	15 (7.5)	60 (30.2)	109 (54.8)	-
[Preventing joint damage/preventing surgery] from [arthritis/IBD]	4 (2.0)	1 (0.5)	10 (5.0)	36 (18.0)	149 (74.5)	-
How quickly biologics would be expected to work	5 (2.5)	6 (3.0)	28 (13.9)	63 (31.3)	99 (49.3)	-
The likelihood that treatment with biologics would work for your child	1 (0.5)	1 (0.5)	3 (1.5)	46 (22.9)	150 (74.6)	-
The overall success rate of treatment with biologics	1 (0.5)	1 (0.5)	7 (3.5)	59 (29.4)	133 (66.2)	-
Your child’s risk of cancer from uncontrolled IBD^c^	2 (1.7)	5 (4.4)	6 (5.2)	33 (28.7)	69 (60.0)	-
Avoiding short-term side effects of biologics	5 (2.5)	14 (7.0)	54 (27.1)	56 (28.1)	54 (27.1)	16 (8.0)
Avoiding long-term side effects of biologics	2 (1.0)	5 (2.5)	17 (8.5)	48 (24.1)	118 (59.3)	9 (4.5)
The risk of tuberculosis (TB) associated with treatment	6 (3.0)	19 (9.5)	34 (17.0)	44 (22.0)	78 (39.0)	19 (9.5)
The effect of treatment on your child’s immune system	1 (0.5)	7 (3.5)	16 (8.0)	62 (31.0)	113 (56.5)	1 (0.5)
The possible risk of cancer associated with treatment	0 (0)	7 (3.5)	15 (7.5)	30 (15.0)	136 (68.0)	12 (6.0)
Avoiding unknown side effects	5 (2.5)	10 (5.1)	43 (21.7)	50 (25.3)	90 (45.5)	-

^a^Indicates this response was not an option

^b^Missing for 52 participants due to their child’s lack of experience with medication side effects

^c^Question only appeared on IBD version of the survey

## Discussion

Parents’ information needs and their influential decision factors are key components of the process they use to make decisions about treatment with TNF-α inhibitors. This survey, designed to assess information needs and influential decision factors for this specific decision, complements existing measures of the decision process that tend to focus narrowly on the interaction between patient and physician [28–30].

Medical decision making has often been conceptualized as involving the patient and the healthcare provider or, in the case of pediatrics, a triad of the patient, parent and provider [23]. Our results suggest that such a model is an oversimplification. Parents seek treatment information from a diversity of people and sources. This finding is consistent with studies in other medical situations, such as parents of pediatric surgical patients and adults undergoing cancer treatment, in which patients used a variety of information sources [14, 31]. However, a German study focused on families of children with rheumatic diseases found that the general practitioner was a source of information for approximately 90 % of parents [16]. The difference in use of general practitioner or primary care provider between that study and ours may be due to differences in the health systems or related to the fact that our study investigated a very specific decision, rather than general information about the child’s condition.

There is an extensive body of literature around health information-seeking behaviors but most studies are not related to a specific decision. For example, some studies have looked at the information seeking behaviors or information needs of parents whose children are seen in the emergency department [17] or focused on medical conditions, [5, 6, 32, 33] rather than a specific treatment decision. We are unaware of other studies that quantify the reasons parents, or other individuals, seek information from sources other than their physician. Participants in our study often sought information to improve their confidence and to understand others’ experiences, rather than due to lack of understanding. This is consistent both with our observational study showing that a large part of the visit is spent on information delivery [10] and with our interview data demonstrating parents’ worry and concern about the decision [7].

Although the disease processes and some of the treatment options differ, [34–36] overall parents’ decision experiences, including information sources used and factors considered, were similar, regardless of whether their child had JIA or IBD. This finding contrasts with our prior qualitative work, likely due to the larger, more diverse sample and reflects the importance of following qualitative work with quantitative studies. The minimal differences seen between diseases likely relate to differences in disease epidemiology [37, 38] and mode of delivery of the most frequently used biologic in each disease (home injection for JIA versus infusion for IBD). The overall similarities allowed us to combine disease groups and provide the benefit of a larger, more generalizable sample for the study. In general, when recalling the influential decision factors, fewer parents seemed to rate treatment logistics, such as cost, location of treatment and route of administration, as very or extremely important compared to the importance given to treatment risks and benefits. This indicates that, for parents of children with either JIA or IBD, the weighing of side effects and efficacy is a key aspect of their treatment decision. The high degree of importance placed by most parents, on most risks and benefits, may be part of the reason parents report struggling with the decision, even when some ultimately see there being no choice [7].

One intervention that may ameliorate some of this struggle is the use of a decision aid, a structured tool designed to provide unbiased information and help parents determine their values related to treatment. Given that few parents reported needing more information or not understanding information from the healthcare provider, a decision aid focused on decisions about TNF-α inhibitors in JIA or IBD should focus less on information delivery and more on helping parents weigh the pros and cons for their family. Specifically, exercises to clarify their values, as related to the treatment decision, may help parents determine treatment goals and weigh the many risks and benefits they consider important to the decision process [39]. The use of decision aids has been associated with improved decision outcomes, specifically increased knowledge and decreased decisional conflict and uncertainty [40]. While our participants did not express a need for more knowledge, the prior qualitative research indicates significant conflict and uncertainty, [7, 41] perhaps due to the difficulty balancing influential decision factors. Future research could also consider how a decision aid might help parents and physicians include the pediatric patient in the decision process. Currently there is only limited evidence regarding the best method of engaging pediatric patients in decision making [42].

The minimal differences seen between parents of children with JIA and parents of children with IBD suggest that the concepts included in our survey could be adapted for other treatment decisions in pediatric rheumatology or other chronic disease settings. Questions in the first domain, Parents’ Information Needs, would require only minimal adaptations to be used for other treatment decisions. However, Influential Decision Factors likely requires a more in-depth understanding of the decision context of interest prior to adaptation.

We undertook a rigorous approach to survey development including extensive testing prior to fielding. Despite this, some individual questions, particularly in the influential decision factors domain, could be considered to demonstrate a floor or ceiling effect. While responses that are skewed limit our ability to determine which items are most important to parents, we feel this reflects the challenges experienced by them and their inability to prioritize decision factors.

Despite following recommended survey procedures [27] that included multiple mailed and phone contacts, our study is limited by its 54.9 % response rate. Furthermore, this process may have introduced selection bias as the research team knew who had responded to the survey. Unfortunately, due to the way one site verified their pool of potential participants, we are unable to determine if non-respondents varied systematically from respondents. Additionally, using medical records and disease registries to identify participants limited our sample to those who had chosen to start TNF-α inhibitors, as the databases contain no indicator of those who decline TNF-α inhibitors. As in any retrospective study, there may be recall bias as parents made this decision up to 2 years prior to receiving the survey. We attempted to minimize such bias by asking parents how old their child was when starting TNF-α inhibitors, to help trigger memories specific to that time. Finally, while this work included only parents, children and adolescents are an integral part of decision making in the pediatric setting. Future work should seek to describe their information needs and influential decision factors.

## Conclusions

This survey, describing decision making about treatment with TNF-α inhibitors, is the first to describe the decision-making process for a specific pediatric chronic condition treatment decision. As such, it offers new insight into the challenges parents’ of children with JIA experience. The information gained through this work provides a framework for understanding parents’ decision-making process and for developing future decision support interventions that may incorporate opportunities to weigh risks and benefits and to include decision participants other than the parent, patient and physician. Furthermore, such interventions may have use beyond decisions about TNF-α inhibitors in JIA as the challenges related to this specific decision are not dissimilar to those in other rheumatologic decisions. In nearly every such decision there are trade-offs to be made, opportunities for families to participate in decision making and currently limited data on how best to support family decision making. Through careful description of the current decision experiences, future interventions will be able to better address parents’ decision-making needs.

## Additional file

Additional file 1: Making Decisions about Biologics Survey. (DOCX 57 kb)

## Abbreviations

JIA: Juvenile idiopathic arthritis
IBD: Inflammatory bowel disease
