# Supplementary material for: Parents’ information needs and influential factors when making decisions about TNF-α inhibitors
Source: Pediatr Rheumatol Online J. 2016 Sep 15;14:53. doi: 10.1186/s12969-016-0113-5 (PMC5024421; doi:10.1186/s12969-016-0113-5)
Supplement: Additional file 1: — Making Decisions about Biologics Survey. (DOCX 57 kb) [file 12969_2016_113_MOESM1_ESM.docx]

**Making Decisions about Biologics**

| *As the parent of a child with arthritis you were selected to participate in this survey because your child started treatment with a biologic medication in the past 2 years.*  *Biologic medications include but are not limited to Remicade (infliximab), Enbrel (entanercept) and Humira (adalimumab). They are designed to block the causes of inflammation in your child’s joints. We will call this group of medications “biologics.” For the rest of the survey, please answer the questions thinking about* ***the first time*** *your child started Remicade (infliximab), Enbrel (entanercept) or Humira (adalimumab). If you have more than one child who has been treated with biologics, please think about the child named in the cover letter.*  *Unless otherwise indicated, please mark only one answer per question.* |
| --- |
| 1. **Does your child have ulcerative colitis or Crohns disease?**   ○ Yes 🡪 Thank you for your time. Please skip to the last page.  ○ No 🡪 Continue to question 2. |
| 1. **Before starting biologics did your child have psoriasis?**   ○ Yes 🡪Thank you for your time. Please skip to the last page.  ○ No 🡪 Continue to question 3. |
| 1. **Exactly how old was your child when they started biologics?** ___________ Years ___________Months |
| 1. **At that time, with which biologic were they treated?**  - Remicade (infliximab) - Enbrel (entanercept) - Humira (adalimumab) |

***This survey is about your decision to start treatment with biologics.***

***When we use the word “biologics” we mean the medication you marked in question 4.***

| *Now we are going to ask you questions about where you got information* ***when you were first deciding about your child taking biologics****. If your child has been on more than one of these medications, please think about the first time you decided to start treatment with one.*   1. **When deciding about treatment with biologics how important was information from each of the following people or resources…** |
| --- |
| \| 1. **Your child’s rheumatology provider?** \| *Didn’t Use* \| Not at all important \| A little important \| Somewhat important \| Very important \| Extremely important \| \| --- \| --- \| --- \| --- \| --- \| --- \| --- \| \| 1. **Your child’s primary care provider?** \| *Didn’t Use* \| Not at all important \| A little important \| Somewhat important \| Very important \| Extremely important \| \| 1. **The rheumatology nurses?** \| *Didn’t use* \| Not at all important \| A little important \| Somewhat important \| Very important \| Extremely important \| \| 1. **Your pharmacist?** \| *Didn’t Use* \| Not at all important \| A little important \| Somewhat important \| Very important \| Extremely important \| \| 1. **Friends and family members?** \| *Didn’t Use* \| Not at all important \| A little important \| Somewhat important \| Very important \| Extremely important \| \| 1. **Parents of children with arthritis?** \| *Didn’t Use* \| Not at all important \| A little important \| Somewhat important \| Very important \| Extremely important \| \| 1. **Materials, such as brochures or videos, from the makers of biologics?** \| *Didn’t Use* \| Not at all important \| A little important \| Somewhat important \| Very important \| Extremely important \| \| 1. **Advertisements?** \| *Didn’t Use* \| Not at all important \| A little important \| Somewhat important \| Very important \| Extremely important \| \| 1. **The internet?** \| *Didn’t Use* \| Not at all important \| A little important \| Somewhat important \| Very important \| Extremely important \| |
| 1. **Other than those listed in question 5, were any other people or resources important sources of information when you were deciding about treatment with biologics?**  - Yes - No   If Yes: **what other kinds of sources did you use?**  **_____________________________________________________________________________________**  **______________________________________________________________________________________** |

| *Parents use sources of information other than their child’s rheumatology provider when making decisions about treatment with biologics. This next group of questions is about why you used different information sources.*   1. **How often did you use sources of information other than your child’s rheumatology provider…**  \| - 1. **To prepare for a rheumatology visit?** \| Never \| Rarely \| Occasionally \| Pretty often \| Very often \|  \| A lot \| \| --- \| --- \| --- \| --- \| --- \| --- \| --- \| --- \| \| - 1. **Because you *did not understand* the information from the rheumatology provider?** \| Never \| Rarely \| Occasionally \| Pretty often \| Very often \|  \|  \| \| - 1. **Because *you wanted more information* than the rheumatology provider gave you?** \| Never \| Rarely \| Occasionally \| Pretty often \| Very often \|  \|  \| \| - 1. **Because *you did not remember* what was said during the visit about treatment with biologics?** \| Never \| Rarely \| Occasionally \| Pretty often \| Very often \|  \|  \| \| - 1. **Because *you wanted to know about other people’s experiences* being treated with biologics?** \| Never \| Rarely \| Occasionally \| Pretty often \| Very often \|  \|  \| \| - 1. **To increase your confidence in your decision?** \| Never \| Rarely \| Occasionally \| Pretty often \| Very often \|  \|  \| |
| --- | --- | --- | --- | --- | --- | --- | --- | --- | --- | --- | --- | --- | --- | --- | --- | --- | --- | --- | --- | --- | --- | --- | --- | --- | --- | --- | --- | --- | --- | --- | --- | --- | --- | --- | --- | --- | --- | --- | --- | --- | --- | --- | --- | --- | --- | --- | --- | --- |
| \| 1. **For parents making decisions about treatment with biologics, when is the best time to get information about treatment options?** *(Check all that apply)*   □ When my child was diagnosed with arthritis  □ When my child’s symptoms got worse  □When my child’s symptoms were not improving  □ Before the doctor’s appointment where we discussed treatment with biologics  □ Other (When else might parents want information? _____________________________________________) \| \| --- \|   *When making a decision about treatment with biologics, most parents need to weigh the pros and cons. This group of questions asks about the importance of these pros and cons when making the decision about your child starting treatment with biologics.*   1. **In making the decision about treatment with biologics, how important was…** |
| \| - 1. **Decreasing the symptoms your child was having at the time?** \| Not at all important \| A little important \| Somewhat important \| Very important \| Extremely important \| \| --- \| --- \| --- \| --- \| --- \| --- \| \| - 1. **Side effects your child had experienced with other kinds of medications?** \| Not at all important \| A little important \| Somewhat important \| Very important \| Extremely important \| \| □ check here if your child has never experienced side effects \| \| \| \| \| \| \| - 1. **The results of medical tests, such as blood work or x-rays?** \| Not at all important \| A little important \| Somewhat important \| Very important \| Extremely important \| \| - 1. **The ways arthritis affects your child’s day to day life?** \| Not at all important \| A little important \| Somewhat important \| Very important \| Extremely important \| \| - 1. **The ways arthritis *treatment* affects your child’s day to day life?** \| Not at all important \| A little important \| Somewhat important \| Very important \| Extremely important \| \| - 1. **Preventing joint damage from JIA?** \| Not at all important \| A little important \| Somewhat important \| Very important \| Extremely important \| \| - 1. **How quickly biologics were expected to work?** \| Not at all important \| A little important \| Somewhat important \| Very important \| Extremely important \| \| **9. (continued) In making the decision about treatment with biologics, how important was…** \| \| \| \| \| \| \| - 1. **How long your child would be treated with biologics?** \| Not at all important \| A little important \| Somewhat important \| Very important \| Extremely important \| \| - 1. **The ability to change your child’s treatment or try other treatments in the future?** \| Not at all important \| A little important \| Somewhat important \| Very important \| Extremely important \| \| - 1. **The likelihood that treatment with biologics would work for your child?** \| Not at all important \| A little important \| Somewhat important \| Very important \| Extremely important \| \| - 1. **The overall success rate of treatment with biologics?** \| Not at all important \| A little important \| Somewhat important \| Very important \| Extremely important \| \| - 1. **The fact that biologics are available only as a shot or IV infusion?** \| Not at all important \| A little important \| Somewhat important \| Very important \| Extremely important \| \| - 1. **The location, such as home or hospital, where your child could receive treatment with biologics?** \| Not at all important \| A little important \| Somewhat important \| Very important \| Extremely important \| \| - 1. **Your child’s age?** \| Not at all important \| A little important \| Somewhat important \| Very important \| Extremely important \| \| - 1. **The out-of-pocket cost of treatment?** \| Not at all important \| A little important \| Somewhat important \| Very important \| Extremely important \|   *The next group of questions asks how important different side effects were in making the decision about treatment with biologics. We will ask about both common and rare side effects.*   1. **In making the decision about treatment with biologics, how important was..** |
| \| - 1. **Avoiding short-term side effects of biologics?** \| Not at all important \| A little important \| Somewhat important \| Very important \| Extremely important \| *Didn’t know about this* \| \| --- \| --- \| --- \| --- \| --- \| --- \| --- \| \| - 1. **Avoiding long-term side effects of biologics?** \| Not at all important \| A little important \| Somewhat important \| Very important \| Extremely important \| *Didn’t know about this* \| \| - 1. **The risk of tuberculosis (TB) associated with treatment?** \| Not at all important \| A little important \| Somewhat important \| Very important \| Extremely important \| *Didn’t know about this* \| \| - 1. **The effect of treatment on your child’s immune system?** \| Not at all important \| A little important \| Somewhat important \| Very important \| Extremely important \| *Didn’t know about this* \| \| - 1. **The possible risk of cancer associated with treatment?** \| Not at all important \| A little important \| Somewhat important \| Very important \| Extremely important \| *Didn’t know about this* \| \| - 1. **Avoiding unknown side effects?** \| Not at all important \| A little important \| Somewhat important \| Very important \| Extremely important \|  \| |
| *The next group of questions is about the process of deciding about treatment with biologics. First we’ll ask about what your child’s rheumatology provider did when you were deciding about treatment with biologics.*   1. When deciding about treatment with biologics, did your child’s rheumatology provider... |

| - 1. Explain the latest medical evidence? | Yes, completely | Yes, somewhat | No |
| --- | --- | --- | --- |
| - 1. Explain the option of doing nothing? | Yes, completely | Yes, somewhat | No |
| - 1. Help you make a decision after considering all the options? | Yes, completely | Yes, somewhat | No |
| - 1. Take the time to understand your goals and concerns? | Yes, completely | Yes, somewhat | No |
| - 1. Explain the benefits of your options? | Yes, completely | Yes, somewhat | No |
| - 1. Explain the risks of your options? | Yes, completely | Yes, somewhat | No |
| - 1. Use language that you understood? | Yes, completely | Yes, somewhat | No |
| - 1. Listen to you? | Yes, completely | Yes, somewhat | No |

| *Now we’ll ask you about how you made the decision about treatment with biologics.* | | | | |  |
| --- | --- | --- | --- | --- | --- |
| 1. Which of these best describes how you made the decision *to have your child start treatment with biologics?* *(choose one)*  - I made the final decision about which treatment my child will receive. - I made the final selection of my child’s treatment after seriously considering my doctor’s opinion. - My doctor and I shared responsibility for deciding which treatment is best for my child. - My doctor made the final decision about which treatment will be used, but seriously considered my opinion. - I left the decision regarding my child’s treatment to my doctor. | | | | | |
| 1. How much involvement in the decision would you have preferred? | | | | | |
| A lot more involvement | A little more involvement | About the same involvement | A little less involvement | A lot less involvement | |
| 1. Was the decision to start biologics made while your child was hospitalized for his/her arthritis?  - Yes - No   *In this section we will ask you questions about how you felt about the decision to start treatment with biologics. Remember, if your child has been on more than one biologic please think about the first time you decided to try one.*  *Considering how you felt when you first made the decision about treatment with biologics, please rate the following statements.* | | | | | |

| 1. I knew which options were available. | Strongly Agree | Agree | Neither Agree nor Disagree | Disagree | Strongly Disagree |
| --- | --- | --- | --- | --- | --- |
| 1. I knew the benefits of each option. | Strongly Agree | Agree | Neither Agree nor Disagree | Disagree | Strongly Disagree |
| 1. I knew the risks and side effects of each option. | Strongly Agree | Agree | Neither Agree nor Disagree | Disagree | Strongly Disagree |
| 1. I was clear about which benefits mattered most to me. | Strongly Agree | Agree | Neither Agree Nor Disagree | Disagree | Strongly Disagree |
| 1. I was clear about which risks and side effects mattered most. | Strongly Agree | Agree | Neither Agree nor Disagree | Disagree | Strongly Disagree |
| 1. I was clear about which was more important (the benefits or the risks and side effects). | Strongly Agree | Agree | Neither Agree nor Disagree | Disagree | Strongly Disagree |
| 1. I had enough support from others to make a choice. | Strongly Agree | Agree | Neither Agree nor Disagree | Disagree | Strongly Disagree |
| 1. I chose without pressure from others. | Strongly Agree | Agree | Neither Agree nor Disagree | Disagree | Strongly Disagree |
| 1. I had enough advice to make a choice. | Strongly Agree | Agree | Neither Agree nor Disagree | Disagree | Strongly Disagree |
| 1. I was clear about the best choice. | Strongly Agree | Agree | Neither Agree nor Disagree | Disagree | Strongly Disagree |
| 1. I felt sure about what to choose. | Strongly Agree | Agree | Neither Agree nor Disagree | Disagree | Strongly Disagree |
| 1. This decision was easy for me to make. | Strongly Agree | Agree | Neither Agree nor Disagree | Disagree | Strongly Disagree |
| 1. I felt I had made an informed choice. | Strongly Agree | Agree | Neither Agree nor Disagree | Disagree | Strongly Disagree |
| 1. My decision showed what is important to me. | Strongly Agree | Agree | Neither Agree nor Disagree | Disagree | Strongly Disagree |
| 1. I expected to stick with my decision. | Strongly Agree | Agree | Neither Agree nor Disagree | Disagree | Strongly Disagree |
| 1. I was satisfied with my decision. | Strongly Agree | Agree | Neither Agree nor Disagree | Disagree | Strongly Disagree |

*Please reflect on the decision you made to start treatment with biologics. Show how strongly you agree or disagree with these statements.*

| 1. It was the right decision. | Strongly Agree | Agree | Neither Agree nor Disagree | Disagree | Strongly Disagree |
| --- | --- | --- | --- | --- | --- |
| 1. I regret the choice that was made. | Strongly Agree | Agree | Neither Agree nor Disagree | Disagree | Strongly Disagree |
| 1. I would go for the same choice if I had to do it over again. | Strongly Agree | Agree | Neither Agree nor Disagree | Disagree | Strongly Disagree |
| 1. The choice did my child a lot of harm. | Strongly Agree | Agree | Neither Agree nor Disagree | Disagree | Strongly Disagree |
| 1. The decision was a wise one. | Strongly Agree | Agree | Neither Agree nor Disagree | Disagree | Strongly Disagree |

| 1. *In general*, when making decisions about your child’s health how do you make the decisions? *(choose one)*   □ I prefer to make the final decision about which treatment my child will receive.  □ I prefer to make the final selection of my child’s treatment after seriously considering my doctor’s opinion.  □ I prefer that my child’s doctor and I share responsibility for deciding which treatment is best for my child.  □ I prefer that my doctor makes the final decision about which treatment will be used, but seriously  considers my opinion.  □ I prefer to leave all decisions regarding my child’s treatment to my doctor.  *These questions ask you about your overall feelings and thoughts during the last month. In each case, please circle how often you felt or thought a certain way.*   1. In the last month, how often have you felt...  \| - 1. That you were unable to control the important things in your life? \| Never \| Almost never \| Sometimes \| Fairly often \| Very often \| \| --- \| --- \| --- \| --- \| --- \| --- \| \| - 1. Confident about your ability to handle your personal problems? \| Never \| Almost never \| Sometimes \| Fairly often \| Very often \| \| - 1. That things were going your way? \| Never \| Almost never \| Sometimes \| Fairly often \| Very often \| \| - 1. Difficulties were piling up so high that you could not overcome them \| Never \| Almost never \| Sometimes \| Fairly often \| Very often \|   *Finally, we’d like to know more about you, your child and your family.* | |
| --- | --- | --- | --- | --- | --- | --- | --- | --- | --- | --- | --- | --- | --- | --- | --- | --- | --- | --- | --- | --- | --- | --- | --- | --- | --- |
| 1. **Exactly how old is your child? __________ Years __________ Months** |  |
| 1. **Is your child male or female?**  - Male - Female |  |
| 1. **How would you describe your child’s race (check all that apply)**  - White - Black/ African American - American Indian/ Alaska Native - Asian - Native Hawaiian/ Other Pacific Islander - Other __________________________ |  |
| 1. **Is your child Hispanic?**   □ Yes  □ No   1. **What is your relationship to the child with arthritis?**  - Mother - Father - Legal Guardian - Other ___________________________ |  |
| 1. **What is the highest level of education you have completed?**  - Did not finish high school or obtain an equivalency degree (GED). - High school graduate or obtained an equivalency degree (GED). - Went to college but did not graduate. - College graduate. - Graduate or professional degree. - Other, please specify _____________________________. |  |
| 1. **Has anyone else in your child’s family been treated with biologics?**  - Yes - No |  |
| 1. **Do you have any other children with a chronic health condition?**  - Yes - No |  |

| 1. **Is there anything else you would like to tell us about your experience making a decision about treatment with biologics?**   _______________________________________________________________________________________________  _______________________________________________________________________________________________  _______________________________________________________________________________________________  _______________________________________________________________________________________________  _______________________________________________________________________________________________  _______________________________________________________________________________________________ |
| --- |
|  |

**Thank you for completing this survey. Please turn the page for instructions on returning the survey.**

Thank you for participating in this study. Even if you only answered question 1 or 2, please fold the survey in half and send it to us in the postage-paid return envelope. If the envelope has been misplaced, please call 513-803-3144 for a new one or mail the survey to:

Cassandra Dodds

Making Decisions about Biologics Survey

3333 Burnet Avenue MLC 7027

Cincinnati OH, 45229
